# Impact of TGEV infection on the pig small intestine

**DOI:** 10.1186/s12985-018-1012-9

**Published:** 2018-06-19

**Authors:** Lu Xia, Yunhan Yang, Jialu Wang, Yuchao Jing, Qian Yang

**Affiliations:** MOE Joint International Research Laboratory of Animal Health and Food Safety, College of Veterinary Medicine, Nanjing Agricultural University, Weigang 1, Nanjing, Jiangsu 210095 People’s Republic of China

**Keywords:** Transmissible gastroenteritis virus, Infection, Pig, Small intestine

## Abstract

**Background:**

Pig diarrhea causes high mortality and large economic losses in the swine industry. Transmissible gastroenteritis virus (TGEV) causes pig diarrhea, with 100% mortality in piglets less than 2 weeks old. No investigation has yet been made of the small intestine of piglets that survived infection by TGEV.

**Methods:**

In this study, we evaluated the impact of TGEV infection on the small intestine of recovered pigs.

**Results:**

Histological analyses showed that TGEV infection led to villi atrophy, and reduced villous height and crypt depth. The number of SIgA positive cells, CD3^+^T cells, and dendritic cells (DCs) in jejunum decreased after TGEV infection in vivo. In contrast, microfold cell (M cell) numbers and cell proliferation increased in infected pigs. TGEV infection also significantly enhanced the mRNA expression levels of cytokine IL-1β, IL-6, TNF-α, IL-10, and TGF-β. Additionally, lower gene copy numbers of *Lactobacillus*, and higher numbers of *Enterobacteriaceae*, were detected in mucosal scraping samples from TGEV-infected pigs.

**Conclusions:**

TGEV infection damages the small intestine, impairs immune functions, and increases pathogenic bacterial loading, all of which may facilitate secondary infections by other pathogens. These findings help quantify the impact of TGEV infection and clarify the pathogenic mechanisms underlying its effects in pigs.

## Background

Pig diarrhea is responsible for considerable economic losses to the swine industry, especially affecting suckling and weaned piglets [1]. In some outbreaks, it causes high morbidity and mortality and survivors exhibit retarded growth [2]. Various enteric pathogens are known to cause pig diarrhea. Porcine transmissible gastroenteritis virus (TGEV), a coronavirus, causes severe diarrhea, vomiting, and dehydration, with mortality rates of 100% in piglets less than 2 weeks old [3]. However, piglets that live more than six to eight days after infection can recover, although they are often stunted. These recovered pigs can spread TGE to uninfected swine for many weeks [4].

The intestinal tract is not only the site of digestion and nutrient absorption, but also acts as a barrier to exclude harmful pathogens and toxins [5]. A healthy gut allows a pig to thrive throughout its lifespan without sickness. Sound animals utilize dietary nutrients efficiently, have better production performance, and generate higher profits for swine producers [6]. The impact of TGEV on the small intestine of growing pigs that survive infection has not been investigated. We therefore evaluated morphological alterations of the small intestine, changes in immune cells in the jejunum, variation of intestinal microflora, and other features in TGEV survivors. The results will help us understand the pathogenesis of TGEV and the overall impact of TGEV infection on swine.

## Methods

### Animals

Six pigs were birthed and maintained in isolated conditions until four weeks of age. The pigs were divided into control and TGEV-infected groups with three pigs per group. Sera from control pigs were confirmed to be negative for TGEV antibodies using an enzyme-linked immunosorbent assay. Piglets were infected with TGEV one day after birth under natural conditions. Piglets that lived more than six to eight days after infection were used in the experiment. The recovered pigs were slaughtered and tissue samples were collected until they were four weeks old. Pigs in both groups were humanely euthanized by intravenous injection of sodium pentobarbital, and duodenum, jejunum, and ileum tissues were immediately sampled. Experimental procedures and animal care protocols were approved by the regulations and guidelines of laboratory animals of Nanjing Agriculture University (Nanjing, China).

### Histological analysis

Tissue samples from the small intestine were fixed in Bouin’s fluid for 48 h at room temperature. After fixation, the samples were sectioned to fit glass slides and then dehydrated in a graded alcohol series (75, 85, 95, 100, and 100% ethanol). The dehydrated blocks were embedded in paraffin, serially sliced into 5-μm-thick sections, and mounted on slides. The sections were dried horizontally on a warming tray overnight at 37 °C, and stained with hematoxylin-eosin (HE) for examination by light microscopy (BH-2, Olympus). Villus height and crypt depth were measured (single-blind) by an observer using computer-assisted morphometry (Image-Pro Plus software). The area of lymphoid follicles in ileal Peyer’s patches (PPs) was also measured.

### Immunohistochemistry

Paraffin sections were de-waxed in xylene and rehydrated in decreasing concentrations of ethanol. Antigen retrieval was performed in citrate buffer (pH 6.0) at 95°C for 30 min. Slides were blocked with 5% BSA (BOSTER, China), and then incubated with anti TGEV N protein antibody, goat anti-pig IgA (Bethyl, USA), rabbit anti-pig CD3 (Abcam, USA), and mouse anti-cytokeratin 18 antibody (Sigma, USA) overnight at 4°C in a humidified chamber. Stained sections were then incubated with biotinylated secondary antibodies for 1 h, and treated with SABC for 1 h. Following each incubation step, slices were washed with PBS 3 times for 5 min per wash. The respective isotypes were used as negative controls. Sections were counterstained with hematoxylin and images were obtained with light microscopy.

### Immunofluorescence staining

For immunofluorescence staining, tissue sections were rinsed and subjected to antigen retrieval as described above. Fc receptors were blocked with 5% bovine serum albumin (BSA) for 1 h. Fixed filters were labeled with combinations of FITC-conjugated Workshop Cluster 3a (SWC3a) (Abcam, USA) and FITC-conjugated histocompatibility leukocyte Ag II-DR (SLA-II-DR) (LSBio, USA), or anti-PCNA (Abcam, USA) overnight at 4°C in a humidified chamber. PBS was used in place of the antibody for the control. Following the overnight treatment, sections were incubated with secondary antibodies for 1 h at room temperature. Cell nuclei were stained with 40, 6-diamidino-2-phenylindole (DAPI) for 5 min, and observed under a confocal laser microscope (Zeiss, Germany). Following each incubation step, slices were washed with PBS 3 times for 5 min per wash.

### Real-time PCR quantification of cytokine expression

RNA was extracted from jejunum tissues and cDNA was synthesized using HiScript II Q RT SuperMix for qPCR (Vazyme, China) according to the manufacturer’s instructions. Quantitative RT-PCR was performed using ChamQ SYBR qPCR Master Mix (Vazyme, China) in a BIOER thermocycler. Primers are shown in Table 1. Data were normalized against GAPDH expression and are expressed as fold differences between control and treated cells using the 2^-ΔΔCT^ method.

**Table 1:** Primers used for RT-PCR

Gene or bacterial target	Primer sequence (5′-3′)	Product size
GAPDH	F: TCATCATCTCTGCCCCTTCT	172 bp
	R: GTCATGAGTCCCTCCACGAT	
IL-1β	F:AGAGGGACATGGAGAAGCGA	209 bp
	R: GCCCTCTGGGTATGGCTTT	
IL-6	F:CCTCGGCAAAATCTCTGCAA	189 bp
	R: TGAAACTCCACAAGACCGGT	
IL-8	F: CCTCATTCCTGTGCTGGTCA	273 bp
	R:TGCAAGTTGAGGCAAGAAGAC	
TGF-β	F: CGCGTGCTAATGGTGGAAAG	132 bp
	R: TGCCCGAGAGAGCAATACAG	
TNF-α	F:GCCCTTCCACCAACGTTTTC	158 bp
	R: TCCCAGGTAGATGGGTTCGT	
IL-10	F: TCTGAGAACAGCTGCATCCAC	112 bp
	R: CGCCCATCTGGTCCTTCGTT	
Total bacteria	F: GTGSTGCAYGGYYGTCGTCA	147 bp
	R: ACGTCRTCCMCNCCTTCCTC	
*Lactobacillus*	F: AGAGGTAGTAACTGGCCTTTA	391 bp
	R: GCGGAAACCTCCCAACA	
*Entero-bacteriaceae*	F: ATGGCTGTCGTCAGCTCGT	385 bp
	R: CCTACTTCTTTTGCAACCCACTC	

### Bacterial quantification by real-time quantitative PCR

Bacterial DNA was extracted from fecal materials and mucosal scrapings using the TIANGEN DNA stool mini kit (TIANGEN, China), following the manufacturer’s guidelines. Primers used to detect total bacteria, *Lactobacillus*, and *Enterobacteriaceae*, are from Heinritz et al. (shown in Table 1) [7]. Standard curves were constructed using purified and quantified PCR products. Serial dilutions were prepared, and 10^2^, 10^3^, 10^4^, and 10^5^ copies of the gene per reaction were used for calibration. Purified PCR amplification products were quantitated using ChamQ SYBR qPCR Master Mix. Quantification was performed in duplicate, and values were averaged.

### Statistical analysis

Results are expressed as means ± standard deviation. Statistical analyses were performed using Student’s t-test. Differences were considered significant at * 0.01 < *p* < 0.05, ** *p* < 0.01.

## Results

### Persistent TGEV infection

**Fig. 1:**
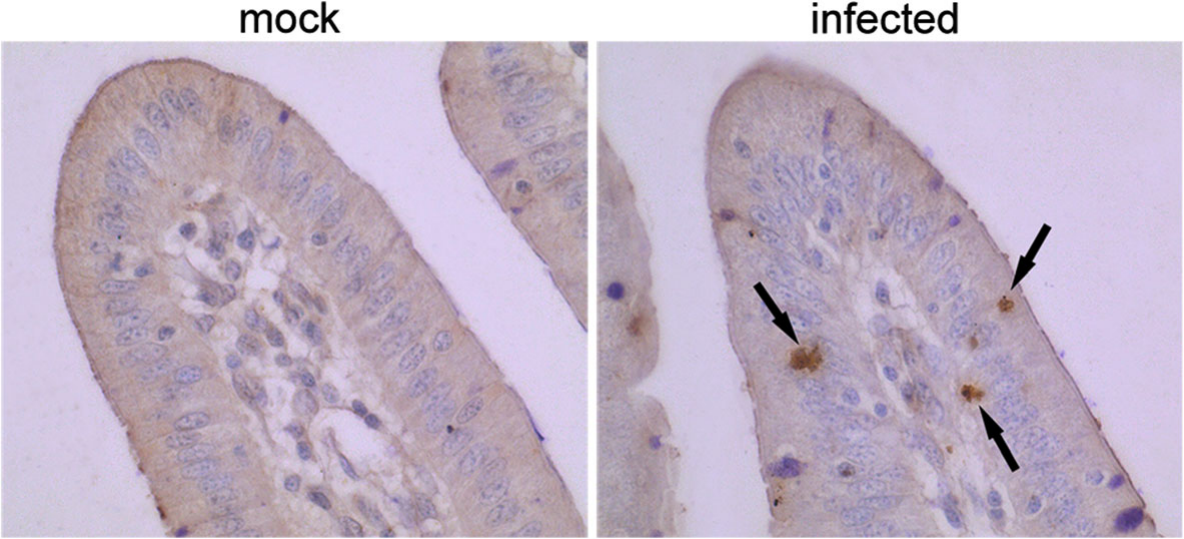
Persistent TGEV infection in recovered pigs. Detection of TGEV infection by immunohistochemistry staining in the jejunum. The TGEV antigen stains brown in the cytoplasm of epithelial cells

To detect whether the recovered pigs persistently infected with TGEV, immunohistochemical staining using an anti TGEV N protein antibody revealed viral antigen. As shown in Fig. 1, paraffin sections from mock-infected pigs had no brown reaction product, while TGEV-positive cells were in the cytoplasm of few scattered epithelial cells or near the luminal surface in TGEV-infected pigs.

### Histopathological analysis

**Fig. 2:**
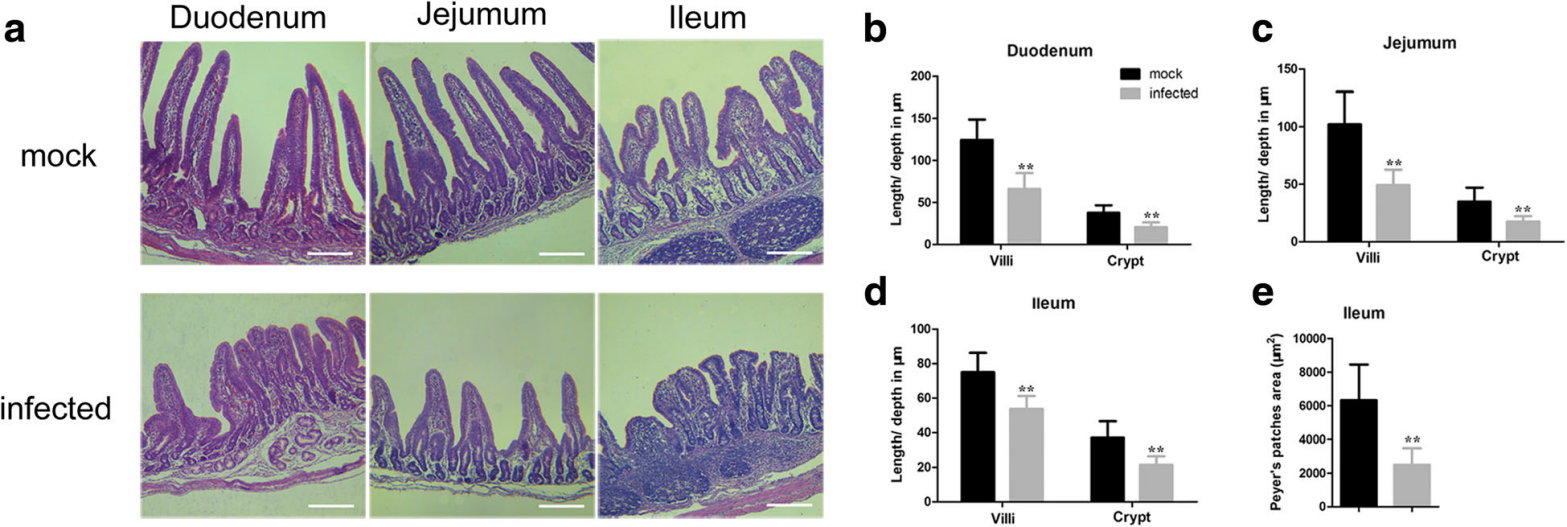
Histological and morphometrical analyses of pig small intestine. **a** HE-stained sections of the duodenum, jejunum and ileum. **b**–**d** Villus height and crypt depth for the small intestine. **e** Area measurements for PPs in ileum. Scale bars: 50 μm. Differences were considered significant at (*) 0.01 < *p* < 0.05, (**) *p* < 0.01

To determine the effects of TGEV infection on the development of porcine gut, the duodenum, jejunum, and ileum of the small intestine were sampled and stained using HE. As shown in Fig. 2a-d, mock-infected pigs had long and slender villi in their small intestines, while TGEV-infected pigs had marked villus shortening, clubbing, and blunting, as well as reduced crypt depth. To determine whether TGEV influences the intestinal immune system, we compared the sizes of ileal PPs (Fig. 2e). Control pigs had significantly larger and more developed PPs than those from infected pigs; the boundaries between partial PPs were also blurred in infected animals.

### Analysis of SIgA positive cells, CD3^+^T cells and DCs in the jejunum

**Fig. 3:**
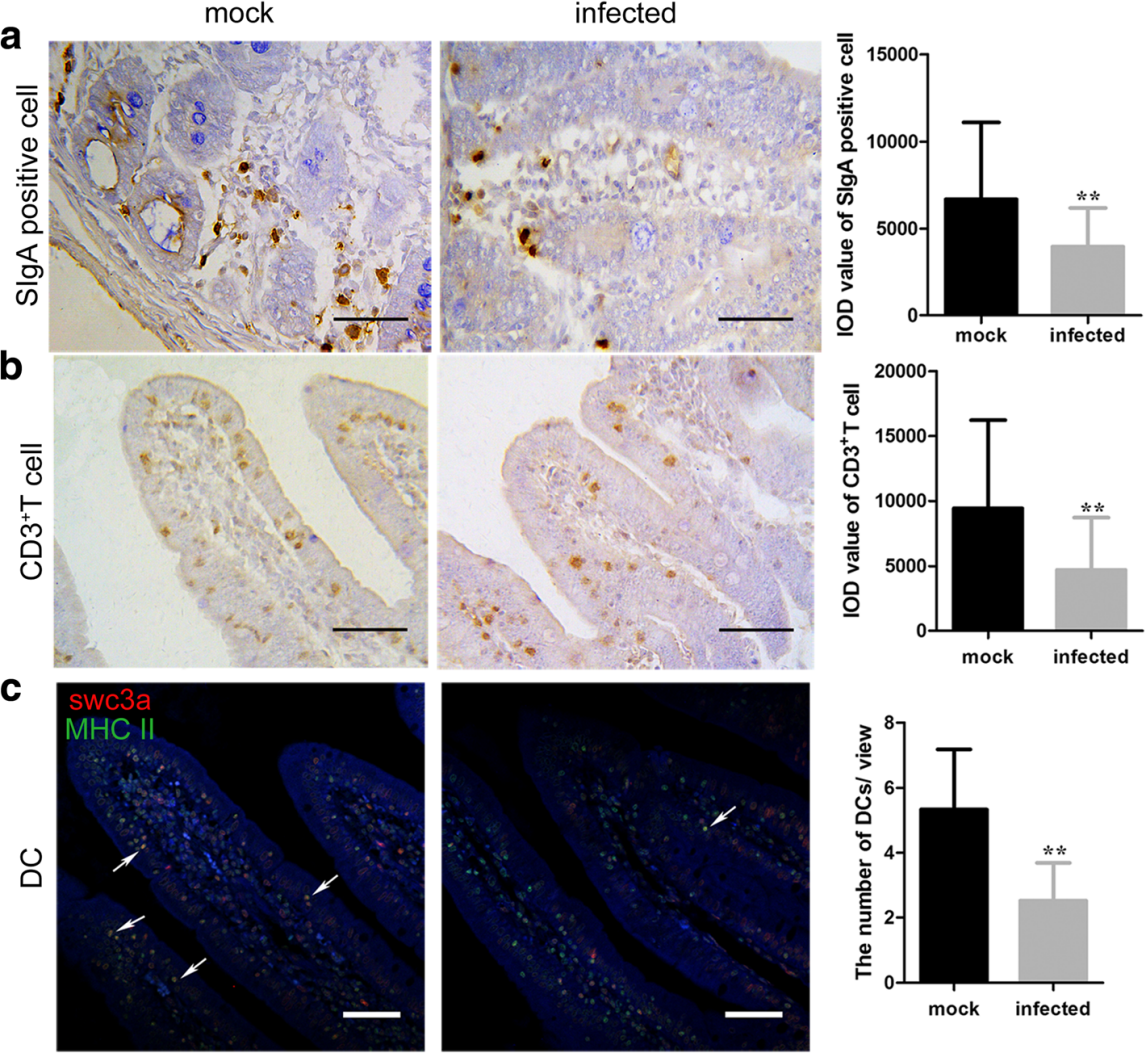
Expression of SIgA positive cells, CD3^+^T cells, DC's. Immunohistochemical staining of SIgA positive cells and CD3^+^T cells, and immunofluorescent staining of DCs in the jejunum. **a** Changes in SIgA positive cells after TGEV infection. **b** Changes in CD3^+^T cells. Positive cells were quantified by densitometry analysis, and were calculated in 10 random fields. IOD: Integrated optical density. **c** Number of SLA-II-DR^+^SWC3a^+^DCs. Scale bars: 50 μm. Differences were considered significant at (*) 0.01 < *p* < 0.05, (**) *p* < 0.01

To examine whether TGEV infection affects immune cells, we determined the number of SIgA positive cells, CD3^+^T cells, and DCs. SIgA positive cells were mainly distributed around the small intestinal glands. Numbers of SIgA positive cells after TGEV infection were significantly lower than in control pigs (Fig. 3a). Immunohistochemical staining (Fig. 3b) showed that CD3^+^T cells were distributed between intestinal epithelial cells and the lamina propria. TGEV infection significantly reduced the number of CD3^+^T cells. DCs were distributed between intestinal epithelial cells and the lamina propria. DCs were less abundant after TGEV infection (Fig. 3c).

### Quantification of M cells and PCNA staining

**Fig. 4:**
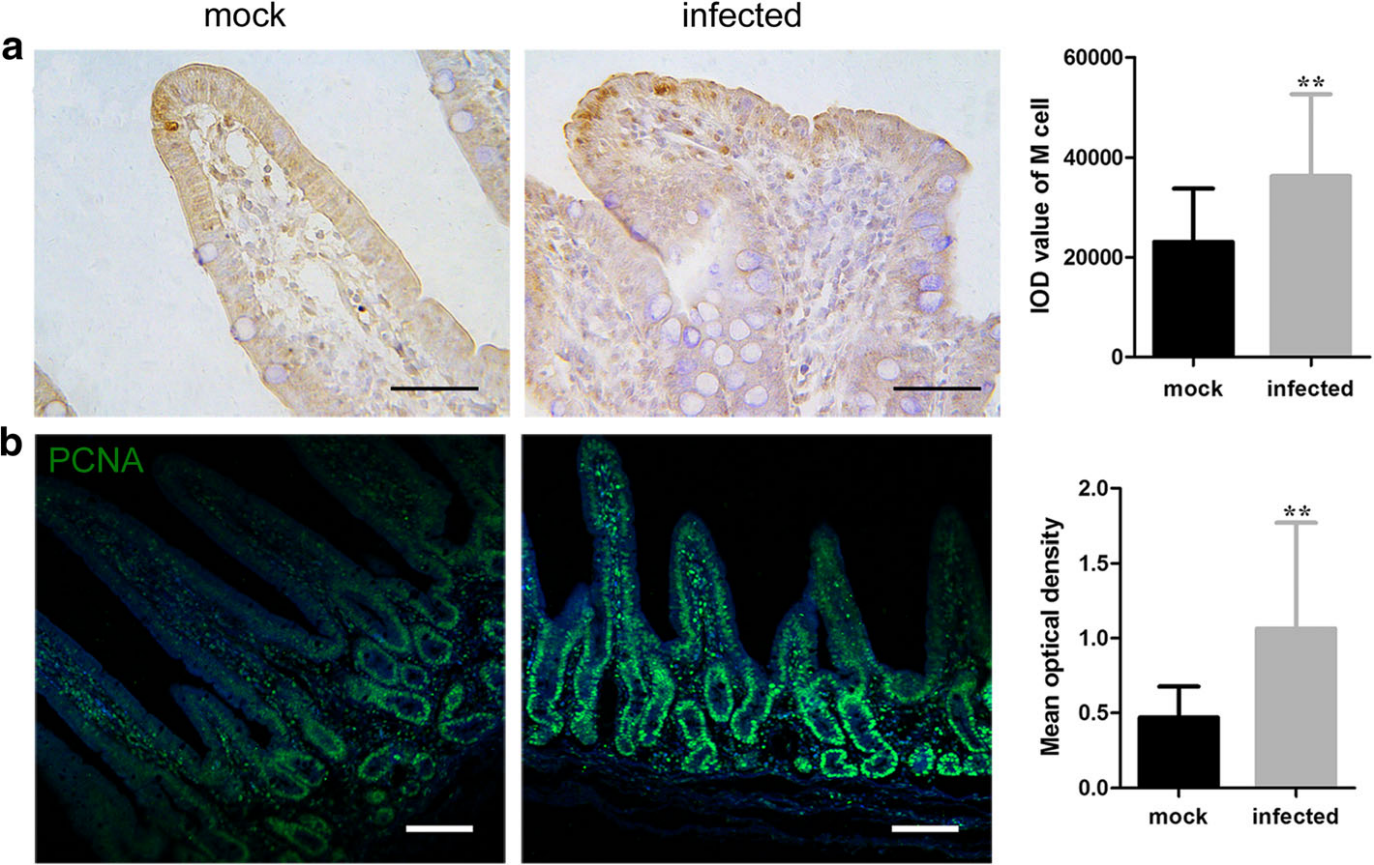
Quantification of M cells and PCNA. Expression of cytokeratin 18, and measurements of intestinal epithelial cell proliferation. **a** Ileal sections were immunostained with anti-cytokeratin 18 monoclonal antibodies. **b** Immunofluorescence staining of proliferation marker PCNA from jejunum tissue. Scale bars: 50 μm in (**a**) and 100 μm in (**b**). Differences were considered significant at (*) 0.01 < *p* < 0.05, (**) *p* < 0.01

M cells are specialized epithelial cells found in the follicle-associated epithelium, and cytokeratin 18 is a specific marker for these intestinal cells [8]. We investigated the expression of cytokeratin 18 in ileal PPs. Fig. 4a shows that M cells are more abundant in the epithelium that lines the villi in infected pigs than in controls. PCNA, a universal marker for proliferating cells, was also examined to determine intestinal epithelial cell proliferation. Infected pigs showed increased jejunal epithelial cell proliferation compared to controls (Fig. 4b).

### TGEV stimulation modulates the expression of cytokines

**Fig. 5:**
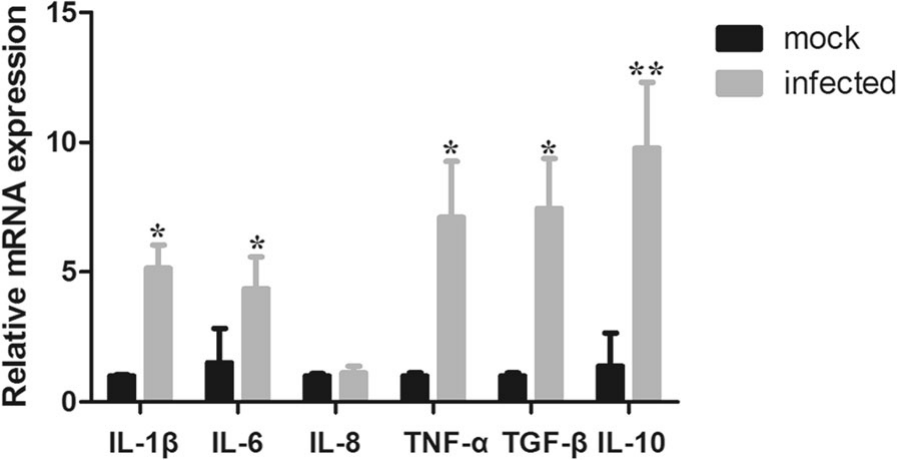
mRNA level of the cytokines. Effect of TGEV infection on cytokines in the jejunum. Relative mRNA levels were measured for the proinflammatory cytokine IL-1β, IL-6, IL-8 and TNF-α, and anti-inflammatory factors IL-10 and TGF-β, using RT-PCR. Differences were considered significant at (*) 0.01 < *p* < 0.05, (**) *p* < 0.01

To investigate whether TGEV modulates cytokine production, we monitored mRNA expression using quantitative RT-PCR. As shown in Fig. 5, TGEV significantly increased expression levels of the proinflammatory cytokinesIL-1β, IL-6, and TNF-α. Additionally, the expression levels of the anti-inflammatory factors IL-10 and TGF-β were also enhanced.

### Effect of TGEV infection on intestinal bacteria

**Fig. 6:**
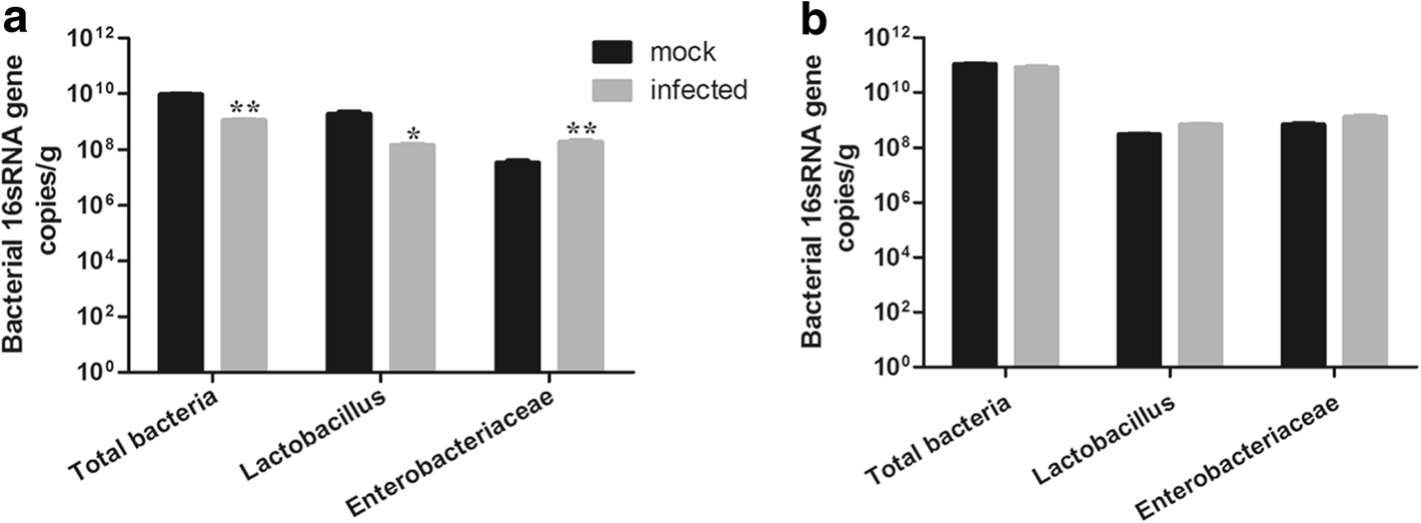
Analysis of total bacteria, *Lactobacillus*, and *Enterobacteriac*eae. Quantitative PCR analysis of gene copy numbers for *Lactobacillus*, *Enterobacteriaceae*, and total bacteria. **a** Bacterial from jejunal mucosal scrapings. **b** Bacteria in feces. Differences were considered significant at (*) 0.01 < *p* < 0.05, (**) *p* < 0.01

Total bacteria, *Lactobacillus*, and *Enterobacteriaceae* were quantified by qPCR. In samples of mucosal scrapings from TGEV-infected pigs, *Lactobacillus* levels declined, while *Enterobacteriaceae* levels increased (Fig. 6a). Bacterial populations in feces did not differ significantly between control and TGEV infected pigs (Fig. 6b).

## Discussion

Although TGEV causes severest pathology in piglets less than 2 weeks old, swine of all ages are susceptible to TGEV infection. Piglets older than 5 weeks frequently survive infection and in adult swine the disease is often mild or unapparent [9]. However, the virus can be readily detected in pigs after they recover, and persists in the lung or gut up to 104 days after infection [10]. Surviving pigs usually show growth retardation and a relatively low rate of weight gain in response to feeding [11]. Since growth retardation may be related to nutrient uptake, we investigated whether TGEV infection impacts the small intestine of surviving piglets.

The small intestine is critical for the digestion, absorption, and metabolism of dietary nutrients. Proper development of the pig gut is not only associated with feeding efficiency, but also with susceptibility to pathogens throughout life. Maldevelopment of the small intestine in TGEV-infected pigs may decrease absorptive capacity and result in a lower feed conversion rate [12]. Our results demonstrated that the cause of impaired growth in TGEV-infected pigs is very likely due to the changes in intestinal morphology and in other characteristics. Julio et al. showed that PEDV infection impairs the performance of surviving pigs, which show increased levels of mortality, reduction in feed conversion ratio, and decreased levels of average daily gain [13]. Aggregated lymphoid follicles, commonly designated PPs, function as immune sensors of the intestine [14]. We found that TGEV-infected pigs have dysplastic PPs, suggesting that their mucosal immune system is immature.

The intestinal immune system is crucial for maintenance of mucosal homeostasis and protection against microbial invasion. SIgA plays an important role in promoting the clearance of antigens and pathogenic microorganisms from the intestinal lumen [15]. The mucosal IgA production is correlated with Th2-dependent cytokines [16]. Most intestinal intraepithelial lymphocytes are CD3^+^T cells, and reside in the intestinal epithelium and subjacent superficial lamina propria, and thus are positioned at the border of the microbial and dietary environment [17]. DCs are specialized antigen-presenting cells and are key regulators of adaptive immune responses, which play important roles in the generation and regulation of the responses to intestinal antigens [18]. We observed fewer IgA secretory cells, CD3^+^T cells, and DCs in TGEV-infected pigs, indicating impaired immune function in the intestinal tract after TGEV infection. The sampling of intestinal pathogens by M cells is important for the induction of an efficient immune response in PPs. However, M cells are also exploited by pathogens as an entry portal for invasion of the host [19]. Certain pathogens, or exposure to inflammatory stimuli, can significantly enhance the density of M cells in the intestine [20]. Our experiments show that TGEV infection apparently increases M cell density. We hypothesize that this reflects a persistent TGEV infection, which induces an inflammatory response.

Intestinal microbiota are important for gastrointestinal function and health. *Lactobacillus* confer beneficial effects on the health of animals, while specific species of *Enterobacteriaceae* are known to be detrimental [21]. Based on PCR analysis, we observed fewer *Lactobacillus* and more *Enterobacteriaceae* in TGEV-infected pigs. Among *Enterobacteriaceae* family, *Escherichia coli* is the most common cause of diarrhea in pigs and piglets [22–24]. Research has shown that animals with signs of intestinal disease have far greater numbers of *Enterobacteriaceae* than do healthy individuals, and that the *Enterobacteriaceae* are linked to intestinal disorders [25, 26].

TGEV infection markedly increased expression levels of the proinflammatory cytokines IL-1β, IL-6, and TNF-α. Proinflammatory cytokine production is important for pathogen clearance, while excessive inflammation can cause tissue damage. The anti-inflammatory master regulator IL-10 balances the proinflammatory signals and limits severe inflammatory responses. However, in some circumstances, elevated IL-10 levels can exhaust antiviral T cells and induce immunosuppression, which enables viral persistence [27]. In addition, proinflammatory cytokines are important factors in chronic inflammatory responses that promote the epithelial-mesenchymal transition (EMT), suggesting that persistent TGEV infection may promote EMT in vivo [28, 29]. TGF-β also functions in immune suppression and plays an important role in viral persistence [30], and TGF-β signaling has been shown to promote IL-10 gene transcription [31].

Intestinal stem cells (ISCs) are rare cells located in intestinal crypts, and are responsible for maintaining the balance between intestinal epithelium damage following injury or inflammation, and epithelial repair by ISC division [32]. Our results show that TGEV infection enhances stem cell proliferation. Mammalian intestinal cells are continuously replenished by stem cells, and are replaced every 2–5 days [5]. It is plausible that TGEV infection induces epithelial loss and thus stimulates the ISC-derived production of new cells to replace damaged epithelial cells [33].

In summary, our results show that TGEV infection damages the small intestine, impairs the intestinal mucosal immune response, and elevates expression levels of inflammatory cytokines. These factors may be associated with chronic inflammatory responses and the persistence of TGEV infection. The increased number of M cells and *Enterobacteriaceae* in mucosal scrapings may be related to secondary infections. This study furthers our understanding of the mechanisms of TGEV infection, and is the first description of how TGEV impacts pigs that survive infection by this virus.

## Conclusions

TGEV infection damages the small intestine, impairs the intestinal mucosal immune response, and increases the number of M cells and *Enterobacteriaceae*, which are related to secondary infections. This is the first investigation of the small intestine in piglets that survived infection by TGEV.

## Abbreviations

DAPI: 4′,6-diamidino-2-phenylindole
DCs: Dendritic cells
EMT: Epithelial-mesenchymal transition
FITC: Fluorescein isothiocyanate
HE: Hematoxylin eosin
IL-1β: Interleukin 1β
IL-6: Interleukin 6
IL-8: Interleukin 8
M cell: Microfold cell
PCNA: Proliferating cell nuclear antigen
PPs: Peyer patches
SABC: Strept avidin biotin complex
SIgA: Secretory Immunoglobulin A
TGEV: Transmissible gastroenteritis virus
TGF-β: Transforming growth factor-β
TNF-α: Tumor necrosis factor α
